# Socioeconomic Status and Knowledge of Cardiovascular Risk Factors: NIPPON DATA2010

**DOI:** 10.2188/jea.JE20170255

**Published:** 2018-03-05

**Authors:** Masayoshi Tsuji, Hisatomi Arima, Takayoshi Ohkubo, Koshi Nakamura, Toshiro Takezaki, Kiyomi Sakata, Nagako Okuda, Nobuo Nishi, Aya Kadota, Tomonori Okamura, Hirotsugu Ueshima, Akira Okayama, Katsuyuki Miura

**Affiliations:** 1 Department of Preventive Medicine and Public Health, Faculty of Medicine, Fukuoka University, Fukuoka, Japan; 2 Department of Hygiene and Public Health, Teikyo University School of Medicine, Tokyo, Japan; 3 Department of Public Health, Hokkaido University, Hokkaido, Japan; 4 Department of International Island and Community Medicine, Kagoshima University, Kagoshima, Japan; 5 Department of Hygiene and Preventive Medicine, Iwate Medical University, Iwate, Japan; 6 Department of Health and Nutrition, University of Human Arts and Sciences, Saitama, Japan; 7 International Center for Nutrition and Information, National Institute of Health and Nutrition, National Institutes of Biomedical Innovation, Health and Nutrition, Tokyo, Japan; 8 Department of Public Health, Shiga University of Medical Science, Shiga, Japan; 9 Center for Epidemiologic Research in Asia, Shiga University of Medical Science, Shiga, Japan; 10 Department of Preventive Medicine and Public Health, Keio University School of Medicine, Tokyo, Japan; 11 Research Institute of Strategy for Prevention, Tokyo, Japan

**Keywords:** socioeconomic status, cardiovascular risk factor, education, household expenditure, general population

## Abstract

**Background:**

The relationship between socioeconomic status (SES) and knowledge of cardiovascular risk factors remains unknown in a general Japanese population.

**Methods:**

Of 8,815 participants from 300 randomly selected areas throughout Japan, 2,467 participants who were free of cardiovascular disease and who provided information on SES in the National Health and Nutrition Survey of Japan 2010 were enrolled in this cross-sectional analysis. SES was classified according to the employment status, length of education, marital and living statuses, and equivalent household expenditure (EHE). Outcomes were ignorance of each cardiovascular risk factor (hypertension, diabetes, hypercholesterolemia, low high-density lipoprotein [HDL] cholesterol, arrhythmia, and smoking) and insufficient knowledge (number of correct answers <4 out of 6).

**Results:**

A short education and low EHE were significantly associated with a greater ignorance of most cardiovascular risk factors. A short education (<10 years) was also associated with insufficient knowledge of overall cardiovascular risk factors: age- and sex-adjusted odds ratios (OR) were 1.92 (95% confidence interval [CI], 1.51–2.45) relative to participants with ≥13 years of education. Low EHE was also associated with insufficient knowledge (age- and sex-adjusted OR 1.24; 95% CI, 1.01–1.51 for the lowest quintile vs the upper 4 quintiles). These relationships remained significant, even after further adjustments for regular exercise, smoking, weekly alcohol consumption, body mass index, hypertension, diabetes mellitus, hypercholesterolemia, and low HDL cholesterol.

**Conclusion:**

Participants with a short education and low EHE were more likely to have less knowledge of cardiovascular risk factors.

## INTRODUCTION

Cardiovascular disease is one of the leading causes of premature death in Japan, as well as other countries worldwide.^1^^,^^2^ The number of deaths due to cardiovascular disease are expected to increase because of the rapid aging of the Japanese society. Therefore, the effective prevention of cardiovascular diseases requires a strategy based on updated knowledge of cardiovascular risk factors in Japan.

Socioeconomic status (SES), which is influenced by factors such as education, income, marital status, living status, and employment status, is associated with risk of cardiovascular disease^3^^,^^4^ or premature death.^5^ Previous studies reported that individuals with low SES were more likely to adopt unhealthy behaviors^6^^–^^8^ and, consequently, be more likely to have cardiovascular risk factors.^9^^,^^10^ The mechanisms underlying the relationship between low SES and unhealthy lifestyles involve a poor family environment^11^^,^^12^ and chronic stress,^13^^,^^14^ while ignorance of cardiovascular risk factors may also play a role in the selection of unhealthy behaviors. To the best of our knowledge, the relationship between SES and knowledge on cardiovascular risk factors has not yet been examined. Therefore, the aim of the present study was to investigate the relationship between SES and knowledge on cardiovascular risk factors in a representative Japanese general population.

## METHODS

### Study population

A prospective cohort study on cardiovascular diseases, the National Integrated Project for Prospective Observation of Non-communicable Disease and its Trends in the Aged 2010 (NIPPON DATA2010), was established in 2010. The study was performed among participants of the National Health and Nutrition Survey of Japan in November 2010 (NHNS2010) and Comprehensive Survey of Living Conditions in June 2010 (CSLC2010), which were conducted by the Ministry of Health, Labour and Welfare of Japan. The details of NHNS2010 and CSLC2010 have been described elsewhere.^15^^–^^19^

In November 2010, 8,815 residents aged 1 year and older from 300 randomly selected districts throughout Japan participated in the dietary survey of NHNS2010. Among 7,229 participants aged ≥20 years, 3,873 had a blood test in NHNS2010, and 2,898 agreed to participate in the baseline survey of NIPPON DATA2010, which also included an electrocardiographic analysis, urinalysis, and a questionnaire on cardiovascular diseases. Trained interviewers obtained informed consent before subject enrollment. The Institutional Review Board of Shiga University of Medical Science (No. 22–29, 2010) approved this study.

This study was a cross-sectional analysis using the baseline survey of NIPPON DATA2010. Of the 2,898 participants, 91 were excluded because it was not possible to merge data from NHNS2010 or CSLC2010 with NIPPON DATA2010 baseline data; 155 with a history of stroke or myocardial infarction were excluded; and 185 with missing data on the employment status, length of education, marital status, living status, or equivalent household expenditure (EHE) were excluded. The remaining 2,467 participants (1,438 women and 1,029 men) were included in the present study.

### Outcomes

Information on knowledge of cardiovascular risk factors was collected using a questionnaire and confirmed by trained interviewers. The participants answered the question ‘Please select all risk factors of stroke or myocardial infarction’, and 6 correct answers were hypertension, diabetes, hypercholesterolemia, low high-density lipoprotein (HDL) cholesterol, arrhythmia, and smoking. Ignorance of each risk factor was used as a binary outcome. Insufficient knowledge of overall cardiovascular risk factors was defined as the number of correct answers of less than 4. The number of correct answers and that weighted by the accuracy rate of each cardiovascular risk factor was also used in sensitivity analyses.

### SES

Information on SES was collected from self-administered questionnaires for NHNS2010 (employment status and annual household income), CSLC2010 (the monthly EHE of 2010 May, month before CSLC2010, and number of household members), and NIPPON DATA2010 (length of education and marital status). EHE was calculated as household expenditure divided by the square root of the number of family members. SES was defined as follows: (i) employment status (employed [including self-employed workers and managers] or unemployed), (ii) length of education (≥13, 10–12, or <10 years), (iii) marital status (ever married [including divorced and widowed] or never married), (iv) living status (living together or living alone), and (v) EHE (upper four quintiles or lowest quintile). Annual household income was also obtained from a self-administered questionnaire for NHNS2010 (<2,000,000 JPY, ≥2,000,000 JPY).

### Other risk factors

Blood pressure (BP) was measured twice with an interval of at least 1 minute, after a minimum of 5 minutes of rest in a seated position using a standard mercury sphygmomanometer with an appropriately-sized cuff on the right arm for NHNS2010. The mean of the two measurements was used in the present study. Casual blood samples were obtained for NHNS2010. If a blood sample was taken after ≥8 hours of fasting, it was defined as a fasting blood sample. Serum was separated and centrifuged soon after blood coagulation. Plasma samples were collected into siliconized tubes containing sodium fluoride and shipped to a central laboratory (SRL, Tokyo, Japan) for analysis. Plasma glucose was measured using the hexokinase UV method, and hemoglobin A1c (HbA1c) using the latex agglutination inhibition assay according to the standardized method of the Japan Diabetes Society (JDS). In the present study, the HbA1c value was converted into the National Glycohemoglobin Standardization Program (NGSP) value using the following formula:

HbA1c(NGSP)(%)=1.02×HbA1c(JDS)(%)+0.25.

Total cholesterol (TC) was measured using enzymatic methods, and standardized by the Center for Disease Control and Prevention/United States Collaborating Center for Reference Method Laboratory Network Research in Blood Lipids.

Public health nurses collected information on smoking (current, ex, or never), alcohol consumption (amount and frequency), and regular exercise (≥30 minutes for ≥2 days a week for more than 1 year) using a standardized questionnaire in NHNS2010. Participants were asked for the usual number of days of the consumption of alcohol beverages per week and the usual amount per day expressed in “go-equivalent” (Japanese unit of alcohol, with 1 go of sake equivalent to 22.1 g of alcohol). Alcohol consumption (g/week) was calculated as the frequency of alcohol intake per week multiplied by the amount of alcohol per day. The height and weight of participants (without shoes) were measured, and body mass index (BMI) was calculated as the ratio of weight (kg) to the square of the height (m).

Participants were classified as hypertensive if they met one of the following criteria^16^: (i) a systolic BP of ≥140 mm Hg, a diastolic BP of ≥90 mm Hg and (ii) the use of antihypertensive medication. Diabetes mellitus was defined as one of the following criteria^16^^,^^20^: (i) fasting plasma glucose level of ≥126 mg/dL; (ii) non-fasting plasma glucose level of ≥200 mg/dL; (iii) HbA1c ≥6.5%; and (iv) use of antidiabetic medications. Hypercholesterolemia was also defined as one of the following^16^^,^^21^: (i) TC ≥220 mg/dL and/or (ii) use of lipid-lowering medication. Low HDL cholesterol was defined as participants with HDL cholesterol <40 mg/dL.^22^

### Statistical analysis

The characteristics of study participants are presented as mean and standard deviation (SD) for continuous variables and as a number and percentage for categorical variables. The relationships among SES and ignorance of each cardiovascular risk factor (hypertension, diabetes mellitus, hypercholesterolemia, low HDL cholesterol, arrhythmia, and smoking) or insufficient knowledge on overall cardiovascular risk factors were assessed using an age- and sex-adjusted logistic regression model. The relationships among SES and the number of correct answers or that weighted by the accuracy rate of each cardiovascular risk factor were assessed using an analysis of covariance (ANCOVA) that included age and sex as covariates. In sensitivity analyses, further adjustments for regular exercise, smoking, alcohol consumption (g/week), and BMI (model 2), as well as with/without hypertension, diabetes mellitus, hypercholesterolemia, and low HDL cholesterol (model 3) were conducted for the outcomes of insufficient knowledge on cardiovascular risk factors and the number of correct answers. The type of house (owned or rented) was obtained from the questionnaire for CSLC2010 and was used as a covariate in analyses of EHE only because expenditure included household rent, but not a mortgage. Differences in the relationships among SES and insufficient knowledge on cardiovascular risk factors or the number of correct answers between subgroups defined by age, sex, and the presence of risk factors (none or having any of hypertension, diabetes, hypercholesterolemia, low HDL cholesterol, or smoking) were assessed by adding interaction terms to the statistical models. *P* < 0.05 was considered to be significant. All statistical analyses were performed using SAS version 9.4 for Windows (SAS Institute Inc., Cary, NC, USA).

## RESULTS

### Participant characteristics

The characteristics of participants in the present study are shown in Table 1. Among 2,467 participants, the mean age was 58.3 years and 1,438 (58.3%) were female. The characteristics of participants by SES are shown in eTable 1. Unemployed participants were older than those who were employed (mean age 65.4 vs 51.8 years), participants with a shorter education were older (<10 years: 68.9 years, 10–12 years: 58.7 years, and ≥13 years: 50.0 years), participants who had ever married were older than those who had never married (60.2 vs 38.8 years), and participants living alone were older than those living together (65.7 vs 57.4 years).

**Table 1.** Characteristics of study participants: NIPPON DATA2010

		All participants (*N* = 2,467)
Age, years		58.3 (15.7)
Sex, women, *n*		1,438 (58.3%)
Regular exercise,^a^ *n*		831 (33.8%)
Smoking	Current smoker, *n*	381 (15.5%)
	Ex-smoker, *n*	460 (18.7%)
Alcohol consumption, g/week		72.1 (133.7)
Body mass index, kg/m^2^		23.1 (3.4)
Systolic blood pressure, mm Hg		132.1 (19.4)
Diastolic blood pressure, mm Hg		79.4 (11.1)
HbA1c (NGSP), %		5.8 (0.8)
Total cholesterol, mg/dL		206.3 (35.1)
HDL cholesterol, mg/dL		62.6 (16.0)
Hypertension,^b^ *n*		1,158 (46.9%)
Diabetes mellitus,^c^ *n*		254 (10.3%)
Hypercholesterolemia,^d^ *n*		1,030 (41.8%)
Low HDL cholesterol,^e^ *n*		181 (7.3%)

HDL, high-density lipoprotein; NGSP, National Glycohemoglobin Standardization Program; NIPPON DATA2010, the National Integrated Project for Prospective Observation of Non-communicable Disease and its Trends in the Aged 2010.

Data is shown as mean (standard deviation [SD]) or *n* (%).

^a^Defined as participants who engaged in exercise of ≥30 minutes for ≥2 days a week for more than one year.

^b^Defined as participants who meet one of the following criteria: (i) Systolic blood pressure/diastolic blood pressure of ≥140 mm Hg/≥90 mm Hg; (ii) Antihypertensive medication.

^c^Defined as participants who meet one of the following criteria: (i) Fasting plasma glucose level of ≥126 mg/dL; (ii) Non-fasting plasma glucose level of ≥200 mg/dL; (iii) Hemoglobin A1c ≥6.5%; (iv) Antidiabetic medication.

^d^Defined as participants who meet one of the following criteria: (i) Total cholesterol ≥220 mg/dL; (ii) Lipid-lowering medication.

^e^Defined as participants who meet the following criterion: (i) High-density lipoprotein cholesterol <40 mg/dL.

### Ignorance of each cardiovascular risk factor

Accuracy rates were 86.5% for hypertension, 45.4% for diabetes mellitus, 73.6% for hypercholesterolemia, 38.3% for low HDL cholesterol, 19.5% for arrhythmia, and 58.4% for smoking. Table 2 and eTable 2 show the frequency and age- and sex-adjusted odds ratios (OR) for ignorance of each cardiovascular risk factor by SES. A shorter education (<10 years) was associated with a higher risk of ignorance of hypertension as a cardiovascular risk factor (17.4%) than an education of ≥13 years (17.4% vs 10.3%; adjusted OR 1.63; 95% confidence interval (CI), 1.15–1.75). Significant differences were also observed in ignorance of other cardiovascular risk factors, except for diabetes (adjusted OR [<10 years vs ≥13 years] 2.45; 95% CI, 1.86–3.22 for insufficient knowledge of hypercholesterolemia; adjusted OR 1.49; 95% CI, 1.16–1.91 for that of low HDL cholesterol; adjusted OR 1.52; 95% CI, 1.19–1.94 for that of arrhythmia, and adjusted OR 1.71; 95% CI, 1.34–2.19 for that of smoking). The lowest quintile group of EHE had more insufficient knowledge of hypertension (17.2% vs 12.6%; adjusted OR 1.43; 95% CI, 1.09–1.87) and that of arrhythmia (55.3% vs 49.3%; adjusted OR 1.31; 95% CI, 1.07–1.60) than the upper four quintile groups. In a sensitivity analysis with the further exclusion of participants with angina pectoris, similar results were observed for the ignorance of each risk factor. There were no marked differences in the relationships between SES and insufficient knowledge on each cardiovascular risk factor between men and women, except for that between EHE and ignorance of smoking (*P* = 0.04 for the interaction).

**Table 2.** Frequency and age- and sex-adjusted odds ratios for insufficient knowledge on each cardiovascular risk factor: NIPPON DATA2010

	Hypertension			Diabetes mellitus			Hypercholesterolemia		
		
	Number of insufficient knowledge/total (%)		OR (95% CI)	Number of insufficient knowledge/total (%)		OR (95% CI)	Number of insufficient knowledge/total (%)		OR (95% CI)
Employment status									
Employed	165/1,293	(12.8)	Reference	721/1,293	(55.8)	Reference	315/1,293	(24.4)	Reference
Unemployed	167/1,174	(14.2)	1.00 (0.77–1.31)	627/1,174	(53.4)	0.94 (0.78–1.13)	336/1,174	(28.6)	1.00 (0.81–1.23)
Length of education									
≥13 years	82/797	(10.3)	Reference	434/797	(54.5)	Reference	143/797	(17.9)	Reference
10–12 years	149/1,090	(13.7)	1.31 (0.98–1.77)	586/1,090	(53.8)	1.03 (0.85–1.24)	288/1,090	(26.4)	1.55 (1.23–1.95)
<10 years	101/580	(17.4)	1.63 (1.15–1.75)	328/580	(56.6)	1.22 (0.96–1.56)	220/580	(37.9)	2.45 (1.86–3.22)
Marital status									
Ever married	301/2,249	(13.4)	Reference	1,229/2,249	(54.7)	Reference	599/2,249	(26.6)	Reference
Never married	31/218	(14.2)	1.44 (0.93–2.24)	119/218	(54.6)	0.90 (0.67–1.22)	52/218	(23.9)	1.28 (0.90–1.83)
Living status									
Living together	288/2,193	(13.1)	Reference	1,198/2,193	(54.6)	Reference	559/2,193	(25.5)	Reference
Living alone	44/274	(16.1)	1.17 (0.83–1.67)	150/274	(54.7)	1.04 (0.80–1.34)	92/274	(33.6)	1.31 (0.99–1.72)
Equivalent household expenditure									
Upper 4 quintiles	249/1,984	(12.6)	Reference	1,077/1,984	(54.3)	Reference	506/1,984	(25.5)	Reference
Lowest quintile	83/483	(17.2)	1.43 (1.09–1.87)	271/483	(56.1)	1.08 (0.89–1.32)	145/483	(30.0)	1.23 (0.98–1.53)



	Low HDL cholesterol			Arrhythmia			Smoking		
		
	Number of insufficient knowledge/total (%)		OR (95% CI)	Number of insufficient knowledge/total (%)		OR (95% CI)	Number of insufficient knowledge/total (%)		OR (95% CI)

Employment status									
Employed	803/1,293	(62.1)	Reference	688/1,293	(53.2)	Reference	511/1,293	(39.5)	Reference
Unemployed	720/1,174	(61.3)	1.09 (0.91–1.32)	557/1,174	(47.4)	1.03 (0.85–1.23)	515/1,174	(43.9)	1.02 (0.85–1.23)
Length of education									
≥13 years	481/797	(60.4)	Reference	407/797	(51.1)	Reference	287/797	(36.0)	Reference
10–12 years	658/1,090	(60.4)	1.08 (0.89–1.31)	534/1,090	(49.0)	1.10 (0.91–1.33)	447/1,090	(41.0)	1.20 (0.98–1.46)
<10 years	384/580	(66.2)	1.49 (1.16–1.91)	304/580	(52.4)	1.52 (1.19–1.94)	292/580	(50.3)	1.71 (1.34–2.19)
Marital status									
Ever married	1,377/2,249	(61.2)	Reference	1,114/2,249	(49.5)	Reference	941/2,249	(41.8)	Reference
Never married	146/218	(67.0)	1.20 (0.87–1.65)	131/218	(60.1)	1.12 (0.82–1.52)	85/218	(39.0)	1.11 (0.81–1.51)
Living status									
Living together	1,351/2,193	(61.6)	Reference	1,110/2,193	(50.6)	Reference	906/2,193	(41.3)	Reference
Living alone	172/274	(62.8)	1.10 (0.85–1.44)	135/274	(49.3)	1.09 (0.84–1.41)	120/274	(43.8)	1.02 (0.79–1.32)
Equivalent household expenditure									
Upper 4 quintiles	1,210/1,984	(61.0)	Reference	978/1,984	(49.3)	Reference	808/1,984	(40.7)	Reference
Lowest quintile	313/483	(64.8)	1.19 (0.96–1.46)	267/483	(55.3)	1.31 (1.07–1.60)	218/483	(45.1)	1.18 (0.97–1.45)

CI, confidence interval; HDL, high-density lipoprotein; OR, odds ratio.

Defined as cardiovascular risk factors of the following criteria: hypertension, diabetes mellitus, hypercholesterolemia, low HDL cholesterol, arrhythmia, and smoking.

Odds ratios were adjusted for age and sex.

### Relationships among SES and insufficient knowledge on cardiovascular disease risk factors

Table 3 and eTable 3 show the relationships among SES and insufficient knowledge of overall cardiovascular risk factors. Of 2,467 participants, 1,160 (47.0%) had insufficient knowledge of overall cardiovascular risk factors (correct answers of less than 4). A shorter education was associated with a higher frequency of insufficient knowledge on cardiovascular risk factors. Age- and sex-adjusted OR were 1.92 (95% CI, 1.51–2.45) for participants with an education of <10 years and 1.23 (95% CI, 1.02–1.49) for those with 10–12 years relative to those with ≥13 years. Low EHE was also associated with increased ignorance of cardiovascular risk factors (age- and sex-adjusted OR 1.24; 95% CI, 1.01–1.51). Similar results were also obtained after further adjustments for other risk factors. There were no relationships between the employment status, marital status, living status, or annual household income and insufficient knowledge on cardiovascular risk factors. There were no marked differences in the relationships among SES and insufficient knowledge on cardiovascular risk factors between subgroups defined by age, sex, and the presence of risk factors (eTable 4).

**Table 3.** Frequency and odds ratio for insufficient knowledge on overall cardiovascular risk factors: NIPPON DATA2010

	Number of insufficient knowledge^a^/total (%)		Model 1		Model 2		Model 3	
		
			OR	(95% CI)	OR	(95% CI)	OR	(95% CI)
Employment status								
Employed	615/1,293	(47.6)	Reference		Reference		Reference	
Unemployed	545/1,174	(46.4)	1.04	(0.86–1.25)	1.11	(0.92–1.34)	1.11	(0.92–1.34)
Length of education								
≥13 years	343/797	(43.0)	Reference		Reference		Reference	
10–12 years	500/1,090	(45.9)	1.23	(1.02–1.49)	1.22	(1.00–1.48)	1.22	(1.01–1.49)
<10 years	317/580	(54.7)	1.92	(1.51–2.45)	1.88	(1.47–2.41)	1.87	(1.46–2.40)
Marital status								
Ever married	1,057/2,249	(47.0)	Reference		Reference		Reference	
Never married	103/218	(47.3)	0.93	(0.68–1.25)	0.95	(0.70–1.30)	0.95	(0.70–1.30)
Living status								
Living together	1,027/2,193	(46.8)	Reference		Reference		Reference	
Living alone	133/274	(48.5)	1.11	(0.86–1.44)	1.13	(0.87–1.46)	1.13	(0.87–1.47)
Equivalent household expenditure								
Upper 4 quintiles	913/1,984	(46.0)	Reference		Reference		Reference	
Lowest quintile	247/483	(51.1)	1.24	(1.01–1.51)	1.22	(1.00–1.49)	1.23	(1.00–1.50)

CI, confidence interval; OR, odds ratio.

^a^Defined as the number of correct answers on cardiovascular risk factors <4.

Model 1 included age and sex.

Model 2 included model 1 plus regular exercise, smoking, weekly alcohol consumption, and body mass index.

Model 3 included model 2 plus hypertension, diabetes mellitus, hypercholesterolemia, low HDL cholesterol, and the type of house (own or rent: in the analysis of equivalent household expenditure only).

### Relationships among SES and the number of correct answers

Table 4 and eTable 5 show the relationships among SES and the number of correct answers on cardiovascular risk factors. The mean (SD) number of correct answers was 3.52 (1.66). The average number of correct answers was smaller among participants with a shorter education (3.19 for <10 years and 3.56 for 10–12 years) than those with ≥13 years of education (3.70). Age- and sex-adjusted differences were significant (−0.61 [95% CI, −0.80 to −0.41] for <10 years and −0.19 [95% CI, −0.35 to −0.03] for 10–12 years). Low EHE (3.31 for the lowest quintile vs 3.57 for the upper 4 quintiles, difference −0.25 [95% CI, −0.42 to −0.09]) was also associated with the number of correct answers. Similar results were obtained after further adjustments for other risk factors. Similar results were also obtained for the outcome of the number of correct answers weighted by the accuracy rate of each cardiovascular risk factor (eTable 6). There were no marked differences in the relationships among SES and the number of correct answers between subgroups defined by age, sex, and the presence of risk factors (eTable 7).

**Table 4.** Average number of correct answers on cardiovascular risk factors and differences: NIPPON DATA2010

	All participants	Mean (95% CI)	Model 1	Model 2	Model 3
		
			Difference (95% CI)	Difference (95% CI)	Difference (95% CI)
Employment status					
Employed	1,293	3.52 (3.43–3.61)	Reference	Reference	Reference
Unemployed	1,174	3.51 (3.42–3.61)	−0.02 (−0.17 to 0.13)	−0.07 (−0.22 to 0.08)	−0.07 (−0.22 to 0.09)
Length of education					
≥13 years	797	3.70 (3.58–3.81)	Reference	Reference	Reference
10–12 years	1,090	3.56 (3.46–3.66)	−0.19 (−0.35 to −0.03)	−0.19 (−0.34 to −0.03)	−0.19 (−0.35 to −0.03)
<10 years	580	3.19 (3.06–3.33)	−0.61 (−0.80 to −0.41)	−0.59 (−0.79 to −0.39)	−0.59 (−0.79 to −0.39)
Marital status					
Ever married	2,249	3.53 (3.45–3.60)	Reference	Reference	Reference
Never married	218	3.37 (3.13–3.61)	−0.16 (−0.41 to 0.10)	−0.16 (−0.42 to 0.09)	−0.17 (−0.42 to 0.09)
Living status					
Living together	2,193	3.53 (3.46–3.60)	Reference	Reference	Reference
Living alone	274	3.40 (3.20–3.59)	−0.13 (−0.35 to 0.08)	−0.15 (−0.36 to 0.06)	−0.16 (−0.37 to 0.06)
Equivalent household expenditure					
Upper 4 quintiles	1,984	3.57 (3.49–3.64)	Reference	Reference	Reference
Lowest quintile	483	3.31 (3.17–3.46)	−0.25 (−0.42 to −0.09)	−0.23 (−0.40 to −0.07)	−0.24 (−0.40 to −0.07)

CI, confidence interval.

Model 1 included age and sex.

Model 2 included model 1 plus regular exercise, smoking, weekly alcohol consumption, and body mass index.

Model 3 included model 2 plus hypertension, diabetes mellitus, hypercholesterolemia, low HDL cholesterol, and the type of house (own or rent: in the analysis of equivalent household expenditure only).

## DISCUSSION

In the present cross-sectional analysis of a representative Japanese general population, some aspects of SES were found to be associated with insufficient knowledge of cardiovascular risk factors. Participants with a shorter education and those with lower EHE were more likely to be ignorant of most cardiovascular risk factors. Similarly, the number of accurate answers on cardiovascular risk factors was smaller among participants with a shorter education and those with lower EHE. In contrast, the employment status, marital status, and living status were not clearly associated with insufficient knowledge of cardiovascular risk factors.

Previous studies demonstrated that individuals with low SES were more likely to adopt unhealthy behaviors, such as smoking, heavy drinking, and physical inactivity.^6^^–^^8^^,^^23^^–^^27^ In the present study, we found relationships between a short education, low EHE, and insufficient knowledge of cardiovascular risk factors. Therefore, the relationship between low SES and an unhealthy lifestyle may partly be attributable to a lack of knowledge of cardiovascular risk factors. Since accurate knowledge of health has been shown to lead to behavioral modifications towards a healthy lifestyle,^28^^,^^29^ the provision of accurate knowledge of cardiovascular risk factors, with a special focus on individuals with a short education and those with low EHE, may be effective for the primary prevention of cardiovascular disease.

The mechanisms underlying the relationships among SES and insufficient knowledge of cardiovascular risk factors remain unclear. A possible mechanism is that there are limited opportunities to learn about cardiovascular risk factors among individuals with a short education. These individuals may also have limited literacy to clearly understand health information. Additionally, individuals with low EHE or a low household income may not be able to afford health information and knowledge.

### Strengths and limitations

To the best of our knowledge, this is the first study to demonstrate relationships among SES and knowledge of cardiovascular risk factors in Japan. The results obtained may be generalizable to all of Japan because the participants were selected from a nationwide survey of a Japanese general population, were older than 20 years, and based on more than 300 randomly selected districts. The present study has some limitations. Since this was a cross-sectional study, a causal relationship between SES and knowledge of cardiovascular risk factors was not clearly defined in the present analysis. A second limitation is the reporting bias on SES because of self-reporting questionnaires. SES may have been affected by the under-reporting of a very high status or over-reporting of a very low status. Furthermore, the results of the present analysis may not be generalizable to other countries in which the education system, lifestyle, and medical system differ from those in Japan.

### Conclusion

Individuals with a short education and those with low EHE were more likely to have less knowledge of cardiovascular risk factors. Since a lack of knowledge of cardiovascular risk factors may lead to the adoption of unhealthy behaviors and subsequent increases in cardiovascular risks, health education targeting these individuals may be effective for the primary prevention of cardiovascular diseases.

## APPENDIX A. SUPPLEMENTARY DATA

eTable 1. Characteristics of study participants by socioeconomic status: NIPPON DATA2010

eTable 2. Frequency and age- and sex-adjusted odds ratios for insufficient knowledge on each cardiovascular risk factor: NIPPON DATA2010

eTable 3. Relationship between annual household income and insufficient knowledge on cardiovascular risk factors: NIPPON DATA2010

eTable 4. Relationships among socioeconomic status and insufficient knowledge on cardiovascular risk factors by age, sex, and the presence of risk factors: NIPPON DATA2010

eTable 5. Relationship between annual household income and the average number of correct cardiovascular risk factors selected by each participant: NIPPON DATA2010

eTable 6. Average number of correct answers weighted by the accuracy rate of each cardiovascular risk factor and adjusted differences: NIPPON DATA2010

eTable 7. Relationships among socioeconomic status and the number of correct answers by age, sex, and the presence of risk factors: NIPPON DATA2010

## References

[1] GBD 2015 Mortality and Causes of Death Collaborators Global, regional, and national life expectancy, all-cause mortality, and cause-specific mortality for 249 causes of death, 1980–2015: a systematic analysis for the Global Burden of Disease Study 2015. Lancet. 2016;388:1459–1544. 10.1016/S0140-6736(16)31012-1 27733281 PMC5388903

[2] *Journal of Health and Welfare Statistics 2016/2017*. Tokyo, Japan: Health, Labour and Welfare Statistics Association; 2016.

[3] Greenland P, Knoll MD, Stamler J, Major risk factors as antecedents of fatal and nonfatal coronary heart disease events. JAMA. 2003;290:891–897. 10.1001/jama.290.7.891 12928465

[4] Magnus P, Beaglehole R The real contribution of the major risk factors to the coronary epidemics: time to end the “only-50%” myth. Arch Intern Med. 2001;161:2657–2660. 10.1001/archinte.161.22.2657 11732929

[5] Stringhini S, Carmeli C, Jokela M, ; LIFEPATH consortium Socioeconomic status and the 25 × 25 risk factors as determinants of premature mortality: a multicohort study and meta-analysis of 1.7 million men and women. Lancet. 2017;389:1229–1237. 10.1016/S0140-6736(16)32380-7 28159391 PMC5368415

[6] Fukuda Y, Nakamura K, Takano T Socioeconomic pattern of smoking in Japan: income inequality and gender and age differences. Ann Epidemiol. 2005;15:365–372. 10.1016/j.annepidem.2004.09.003 15840550

[7] Power C, Graham H, Due P, The contribution of childhood and adult socioeconomic position to adult obesity and smoking behaviour: an international comparison. Int J Epidemiol. 2005;34:335–344. 10.1093/ije/dyh394 15659473

[8] Kivimäki M, Lawlor DA, Davey Smith G, Socioeconomic position, co-occurrence of behavior-related risk factors, and coronary heart disease: the Finnish Public Sector study. Am J Public Health. 2007;97:874–879. 10.2105/AJPH.2005.078691 17395837 PMC1854863

[9] Fukuda Y, Hiyoshi A Associations of household expenditure and marital status with cardiovascular risk factors in Japanese adults: analysis of nationally representative surveys. J Epidemiol. 2013;23:21–27. 10.2188/jea.JE20120021 23208515 PMC3700239

[10] Colhoun HM, Hemingway H, Poulter NR Socio-economic status and blood pressure: an overview analysis. J Hum Hypertens. 1998;12:91–110. 10.1038/sj.jhh.1000558 9504351

[11] Loucks EB, Lynch JW, Pilote L, Life-course socioeconomic position and incidence of coronary heart disease: the Framingham Offspring Study. Am J Epidemiol. 2009;169:829–836. 10.1093/aje/kwn403 19179358 PMC2727217

[12] Bartley M, Plewis I Accumulated labour market disadvantage and limiting long-term illness: data from the 1971–1991 Office for National Statistics’ Longitudinal Study. Int J Epidemiol. 2002;31:336–341. 11980794

[13] Kondo N Socioeconomic disparities and health: impacts and pathways. J Epidemiol. 2012;22:2–6. 10.2188/jea.JE20110116 22156290 PMC3798573

[14] Landsbergis PA, Schnall PL, Pickering TG, Warren K, Schwartz JE Lower socioeconomic status among men in relation to the association between job strain and blood pressure. Scand J Work Environ Health. 2003;29:206–215. 10.5271/sjweh.723 12828390

[15] Kadota A, Okuda N, Ohkubo T, The National Integrated Project for Prospective Observation of Non-communicable Disease and its Trends in the Aged 2010 (NIPPON DATA2010): objectives, design, and population characteristics. J Epidemiol. 2018;28(Suppl 3):S2–S9. 10.2188/jea.JE20170240 PMC5825689 29503381

[16] Satoh A, Arima H, Ohkubo T, ; NIPPON DATA2010 Research Group Associations of socioeconomic status with prevalence, awareness, treatment, and control of hypertension in a general Japanese population. J Hypertens. 2017;35:401–408. 10.1097/HJH.0000000000001169 28005709

[17] Ikeda N, Takimoto H, Imai S, Miyachi M, Nishi N Data resource profile: the Japan National Health and Nutrition Survey (NHNS). Int J Epidemiol. 2015;44:1842–1849. 10.1093/ije/dyv152 26239276

[18] Ikeda N, Shibuya K, Hashimoto H Improving population health measurement in national household surveys: a simulation study of the sample design of the comprehensive survey of living conditions of the people on health and welfare in Japan. J Epidemiol. 2011;21:385–390. 10.2188/jea.JE20100102 21841351 PMC3899438

[19] Katanoda K, Matsumura Y National Nutrition Survey in Japan—its methodological transition and current findings. J Nutr Sci Vitaminol. 2002;48:423–432. 10.3177/jnsv.48.423 12656220

[20] Committee of the Japan Diabetes Society on the Diagnostic Criteria of Diabetes Mellitus, Seino Y, Nanjo K, Tajima N, Report of the committee on the classification and diagnostic criteria of diabetes mellitus. J Diabetes Investig. 2010;1:212–228. 10.1111/j.2040-1124.2010.00074.x 24843435 PMC4020724

[21] Teramoto T, Sasaki J, Ueshima H, ; Japan Atherosclerosis Society (JAS) Committee for Epidemiology and Clinical Management of Atherosclerosis Diagnostic criteria for dyslipidemia. Executive summary of Japan Atherosclerosis Society (JAS) guideline for diagnosis and prevention of atherosclerotic cardiovascular diseases for Japanese. J Atheroscler Thromb. 2007;14:155–158. 10.5551/jat.E537 17827859

[22] Nagai M, Ohkubo T, Kadota A, ; NIPPON DATA2010 Research Group Awareness of risk factors as causes of cardiovascular disease in the general population and awareness in those with and without these risk factors: NIPPON DATA2010. Jpn J Cardiovasc Dis Prev. 2016;51:166–175.

[23] Kagamimori S, Gaina A, Nasermoaddeli A Socioeconomic status and health in the Japanese population. Soc Sci Med. 2009;68:2152–2160. 10.1016/j.socscimed.2009.03.030 19375838

[24] Blane D, Hart CL, Smith GD, Association of cardiovascular disease risk factors with socioeconomic position during childhood and during adulthood. BMJ. 1996;313:1434–1438. 10.1136/bmj.313.7070.1434 8973230 PMC2352956

[25] Laaksonen M, Rahkonen O, Karvonen S, Lahelma E Socioeconomic status and smoking: analysing inequalities with multiple indicators. Eur J Public Health. 2005;15:262–269. 10.1093/eurpub/cki115 15755781

[26] Langenberg C, Kuh D, Wadsworth ME, Brunner E, Hardy R Social circumstances and education: life course origins of social inequalities in metabolic risk in a prospective national birth cohort. Am J Public Health. 2006;96:2216–2221. 10.2105/AJPH.2004.049429 17077402 PMC1698170

[27] Wardle J, Waller J, Jarvis MJ Sex differences in the association of socioeconomic status with obesity. Am J Public Health. 2002;92:1299–1304. 10.2105/AJPH.92.8.1299 12144988 PMC1447234

[28] Jepson RG, Harris FM, Platt S, Tannahill C The effectiveness of interventions to change six health behaviours: a review of reviews. BMC Public Health. 2010;10:538. 10.1186/1471-2458-10-538 20825660 PMC2944371

[29] Mosca L, Mochari H, Christian A, National study of women’s awareness, preventive action, and barriers to cardiovascular health. Circulation. 2006;113:525–534. 10.1161/CIRCULATIONAHA.105.588103 16449732

