# Supplementary material for: Socioeconomic Status and Knowledge of Cardiovascular Risk Factors: NIPPON DATA2010
Source: J Epidemiol. 2018 Mar 5;28(Suppl 3):S46–52. doi: 10.2188/jea.JE20170255 (PMC5825694; doi:10.2188/jea.JE20170255)
Supplement: Supplementary file 1 [file je-28-S046-s001.pdf]

eTable 1. Characteristics of study participants by socioeconomic status: NIPPON DATA2010

		Employment status			Length of education				Marital status			Living status			Equivalent household expenditure		
		Employed (n=1,293)	Unemployed (n=1,174)		≥13 years (n=797)	10-12 years (n=1,090)	<10 years (n=580)		Ever married (n=2,249)	Never married (n=218)		Living together (n=2,193)	Living alone (n=274)		Upper 4 quintiles (n=1,984)	Lowest quintile (n=483)	
Age, years		51.8 (14.3)	65.4 (14.0)	*	50.0 (15.1)	58.7 (14.6)	68.9 (11.2)	*	60.2 (14.5)	38.8 (14.3)	*	57.4 (15.5)	65.7 (15.0)	*	58.0 (15.6)	59.5 (16.1)	
Sex, women, n		616 (47.6%)	822 (70.0%)	†	448 (56.2%)	658 (60.4%)	332 (57.2%)		1333 (59.3%)	105 (51.8%)	†	1261 (57.5%)	177 (64.6%)	†	1154 (58.2%)	284 (58.8%)	
Regular exercise, n		324 (25.1%)	507 (43.3%)	†	235 (29.5%)	380 (35.0%)	216 (37.3%)	†	776 (34.6%)	55 (25.4%)	†	711 (32.5%)	120 (43.8%)	†	678 (34.2%)	153 (31.8%)	
Smoking	Current smoker, n	281 (21.8%)	100 (8.5%)	†	107 (13.4%)	182 (16.8%)	92 (15.9%)		326 (14.5%)	55 (25.2%)	†	338 (15.4%)	43 (15.8%)		303 (15.3%)	78 (16.2%)	
	Ex-smoker, n	247 (19.1%)	213 (18.2%)		143 (18.0%)	201 (18.5%)	116 (20.0%)		437 (19.5%)	23 (10.6%)		419 (19.1%)	41 (15.0%)		364 (18.4%)	96 (19.9%)	
Alcohol consumption, g/week		95.0 (152.5)	46.9 (103.7)	*	70.6 (124.2)	76.1 (140.8)	66.7 (132.7)		73.7 (135.7)	56.1 (109.9)		72.7 (133.6)	67.6 (134.6)		75.1 (139.2)	60.1 (107.9)	*
Body mass index, kg/m^2		23.1 (3.5)	23.1 (3.4)		22.7 (3.4)	23.1 (3.3)	23.7 (3.5)	*	23.1 (3.3)	22.8 (4.4)		23.1 (3.4)	23.4 (3.3)		23.1 (3.4)	23.2 (3.6)	
Systolic blood pressure, mm Hg		128.9 (18.3)	135.6 (20.0)	*	125.8 (18.0)	133.0 (19.2)	138.9 (18.9)	*	133.1 (19.3)	121.0 (17.0)	*	131.2 (19.2)	138.9 (20.1)	*	131.8 (19.3)	133.0 (20.0)	
Diastolic blood pressure, mm Hg		79.5 (11.0)	79.2 (11.1)		78.3 (10.9)	79.8 (10.9)	79.9 (11.4)	*	79.7 (11.0)	76.1 (11.7)	*	79.2 (11.0)	80.7 (11.4)	*	79.4 (11.0)	79.2 (11.4)	
HbA1c (NGSP), %		5.7 (0.8)	5.9 (0.8)	*	5.6 (0.7)	5.8 (0.8)	5.9 (0.8)	*	5.8 (0.8)	5.5 (1.0)	*	5.8 (0.7)	5.9 (1.0)	*	5.8 (0.8)	5.8 (0.8)	
Total cholesterol, mg/dL		205.6 (34.1)	207.1 (36.1)		204.6 (35.5)	209.2 (35.2)	203.3 (33.8)	*	206.8 (34.8)	201.4 (37.4)	*	206.0 (34.9)	209.0 (36.1)		206.7 (34.8)	204.7 (36.0)	
HDL cholesterol, mg/dL		63.0 (15.9)	62.1 (16.0)		64.3 (16.0)	62.7 (16.0)	60.1 (15.6)	*	62.7 (15.8)	61.8 (17.5)		62.5 (15.9)	63.1 (16.7)		62.9 (16.0)	61.2 (15.6)	*
Hypertension, n		505 (39.1%)	653 (55.6%)	†	261 (32.8%)	524 (48.1%)	373 (64.3%)	†	1107 (49.2%)	51 (23.4%)	†	977 (44.6%)	181 (66.1%)	†	914 (46.1%)	244 (50.5%)	
Diabetes mellitus, n		108 (8.4%)	146 (12.4%)	†	47 (5.9%)	119 (10.9%)	88 (15.2%)	†	246 (10.9%)	8 (3.7%)	†	209 (9.5%)	45 (16.4%)	†	207 (10.4%)	47 (9.7%)	
Hypercholesterolemia, n		481 (37.2%)	549 (46.8%)	†	285 (35.8%)	485 (44.5%)	260 (44.8%)	†	968 (43.0%)	62 (28.4%)	†	901 (41.1%)	129 (47.1%)		820 (41.3%)	210 (43.5%)	
Low HDL cholesterol, n		81 (6.3%)	100 (8.5%)	†	47 (5.9%)	80 (7.3%)	54 (9.3%)		158 (7.0%)	23 (10.6%)		158 (7.2%)	23 (8.4%)		142 (7.2%)	39 (8.1%)	

Data is shown as mean (standard deviation [SD]) or n (%).

HDL, high-density lipoprotein; NGSP, National Glycohemoglobin Standardization Program; NIPPON DATA2010, the National Integrated Project for Prospective Observation of Non-communicable Disease and its Trends in the Aged 2010.

* P <0.05 calculated by the *t*-test, Wilcoxon signed-rank test, or an analysis of variance by continuous variables.

† P <0.05 calculated by the chi-squared test by categorical variables.

eTable 2. Frequency and age- and sex-adjusted odds ratios for insufficient knowledge on each cardiovascular risk factor: NIPPON DATA2010

	Hypertension			Diabetes mellitus			Hypercholesterolemia		
	Number of insufficient knowledge/total (%)		OR (95% CI)	Number of insufficient knowledge/total (%)		OR (95% CI)	Number of insufficient knowledge/total (%)		OR (95% CI)
Annual household income									
≥2,000,000 Japanese yen	225 / 1,818	(12.4)	Reference	1,005 / 1,818	(55.3)	Reference	435 / 1,818	(23.9)	Reference
<2,000,000 Japanese yen	73 / 420	(17.4)	1.38	217 / 420	(51.7)	0.89	149 / 420	(35.5)	1.55
			(1.02–1.85)			(0.72–1.11)			(1.23–1.96)

continued	Low HDL cholesterol			Arrhythmia			Smoking		
	Number of insufficient knowledge/total (%)		OR (95% CI)	Number of insufficient knowledge/total (%)		OR (95% CI)	Number of insufficient knowledge/total (%)		OR (95% CI)
	1,121 / 1,818	(61.7)	Reference	915 / 1,818	(50.3)	Reference	723 / 1,818	(39.8)	Reference
	260 / 420	(61.9)	1.06	213 / 420	(50.7)	1.19	196 / 420	(46.7)	1.23
			(0.84–1.32)			(0.95–1.48)			(0.98–1.53)

CI, confidence interval; OR, odds ratio.

The model included age and sex.

eTable 3. Relationship between annual household income and insufficient knowledge on cardiovascular risk factors: NIPPON DATA2010

	Number of insufficient knowledge[a]/total	(%)	Model 1		Model 2		Model 3	
			OR	95% CI	OR	95% CI	OR	95% CI
Annual household income								
≥2,000,000 Japanese yen	837 / 1,818	(46.0)	Reference		Reference		Reference	
<2,000,000 Japanese yen	204 / 420	(48.6)	1.15	(0.93–1.43)	1.16	(0.93–1.44)	1.17	(0.93–1.47)

CI, confidence interval; OR, odds ratio.

[a] Defined as the number of correct answers on cardiovascular risk factors <4.

Model 1 included age and sex.

Model 2 included model 1 plus regular exercise, smoking, weekly alcohol consumption, and body mass index.

Model 3 included model 2 plus hypertension, diabetes mellitus, hypercholesterolemia, low HDL cholesterol, and the square root of the number of household members.

eTable 4. Relationships among socioeconomic status and insufficient knowledge on cardiovascular risk factors by age, sex, and the presence of risk factors: NIPPON DATA2010

	Age					Sex					Presence of risk factors				
	<65 years (n=1,453)		≥65 years (n=1,014)		P for hetero-geneity	Women (n=1,438)		Men (n=1,029)		P for hetero-geneity	None (n=633)		Having any risk factor (n=1,832)		P for hetero-geneity
	OR	95% CI	OR	95% CI		OR	95% CI	OR	95% CI		OR	95% CI	OR	95% CI	
Employment status															
Employed	Reference		Reference			Reference		Reference			Reference		Reference		
Unemployed	0.93	(0.73–1.19)	0.97	(0.73–1.29)	0.887	1.12	(0.89–1.41)	0.90	(0.66–1.22)	0.533	0.89	(0.63–1.26)	1.09	(0.87–1.35)	0.241
Length of education															
≥13 years	Reference		Reference			Reference		Reference			Reference		Reference		
10–12 years	1.15	(0.92–1.43)	1.14	(0.79–1.66)		1.14	(0.88–1.48)	1.38	(1.04–1.84)		1.43	(1.00–2.02)	1.17	(0.93–1.47)	
<10 years	1.77	(1.25–2.51)	1.61	(1.11–2.34)	0.838	1.96	(1.41–2.73)	1.86	(1.30–2.66)	0.761	2.37	(1.34–4.18)	1.81	(1.38–2.38)	0.818
Marital status															
Ever married	Reference		Reference			Reference		Reference			Reference		Reference		
Never married	1.04	(0.78–1.40)	0.65	(0.16–2.74)	0.530	1.10	(0.72–1.69)	0.79	(0.51–1.21)	0.196	0.98	(0.60–1.59)	0.89	(0.60–1.32)	0.517
Living status															
Living together	Reference		Reference			Reference		Reference			Reference		Reference		
Living alone	0.99	(0.65–1.49)	1.13	(0.81–1.57)	0.620	1.15	(0.83–1.60)	1.10	(0.73–1.67)	0.950	1.21	(0.64–2.29)	1.10	(0.83–1.45)	0.868
Equivalent household expenditure															
Upper 4 quintiles	Reference		Reference			Reference		Reference			Reference		Reference		
Lowest quintile	1.10	(0.84–1.43)	1.42	(1.05–1.92)	0.216	1.45	(1.12–1.89)	0.99	(0.73–1.35)	0.070	1.28	(0.85–1.94)	1.22	(0.97–1.53)	0.827

CI, confidence interval; OR, odds ratio.

"Having any risk factor" was defined as having at least one of hypertension, diabetes, hypercholesterolemia, low HDL cholesterol, or smoking.

Models include age (except for age groups) and sex (except for gender groups).

eTable 5. Relationship between annual household income and the average number of correct cardiovascular risk factors selected by each participant: NIPPON DATA2010

	Total participants	Mean (95% CI)		Model 1		Model 2		Model 3	
				Difference (95% CI)		Difference (95% CI)		Difference (95% CI)	
Annual household income									
≥2,000,000 Japanese yen	1818	3.57	(3.49–3.64)	Reference		Reference		Reference	
<2,000,000 Japanese yen	420	3.36	(3.20–3.52)	−0.21	(−0.39 to −0.03)	−0.21	(−0.39 to −0.03)	−0.26	(−0.44 to −0.07)

CI, confidence interval.

Model 1 included age and sex.

Model 2 included model 1 plus regular exercise, smoking, weekly alcohol consumption, and body mass index.

Model 3 included model 2 plus hypertension, diabetes mellitus, hypercholesterolemia, low HDL cholesterol, and the square root of the number of household members.

eTable 6. Average number of correct answers weighted by the accuracy rate of each cardiovascular risk factor and adjusted differences: NIPPON DATA2010

	All participants	Mean (95% CI)		Model 1		Model 2		Model 3	
				Difference (95% CI)		Difference (95% CI)		Difference (95% CI)	
Employment status									
Employed	1293	2.24	(2.19–2.29)	Reference		Reference		Reference	
Unemployed	1174	2.22	(2.16–2.27)	−0.01	(−0.10 to 0.08)	−0.04	(−0.13 to 0.05)	−0.04	(−0.13 to 0.05)
Length of education									
≥13 years	797	2.35	(2.29–2.42)	Reference		Reference		Reference	
10–12 years	1090	2.25	(2.19–2.30)	−0.12	(−0.21 to −0.03)	−0.12	(−0.21 to −0.03)	−0.12	(−0.21 to −0.03)
<10 years	580	2.02	(1.95–2.10)	−0.36	(−0.48 to −0.25)	−0.35	(−0.47 to −0.24)	−0.35	(−0.46 to −0.23)
Marital status									
Ever married	2249	2.24	(2.20–2.28)	Reference		Reference		Reference	
Never married	218	2.13	(2.00–2.27)	−0.10	(−0.24 to 0.04)	−0.11	(−0.25 to 0.04)	−0.11	(−0.25 to 0.04)
Living status									
Living together	2193	2.24	(2.20–2.28)	Reference		Reference		Reference	
Living alone	274	2.14	(2.03–2.26)	−0.09	(−0.21 to 0.04)	−0.09	(−0.21 to 0.03)	−0.09	(−0.22 to 0.03)
Equivalent household expenditure									
Upper 4 quintiles	1984	2.26	(2.22–2.30)	Reference		Reference		Reference	
Lowest quintile	483	2.11	(2.02–2.19)	−0.15	(−0.24 to −0.05)	−0.14	(−0.23 to −0.04)	−0.14	(−0.24 to −0.05)

CI, confidence interval.

Model 1 included age and sex.

Model 2 included model 1 plus regular exercise, smoking, weekly alcohol consumption, and body mass index.

Model 3 included model 2 plus hypertension, diabetes mellitus, hypercholesterolemia, low HDL cholesterol, and the type of house (own or rent: in the analysis of equivalent household expenditure only).

eTable 7. Relationships among socioeconomic status and the number of correct answers by age, sex, and the presence of risk factors: NIPPON DATA2010

	Age					Sex					Presence of risk factors				
	<65 years (n=1,453) Difference (95% CI)		≥65 years (n=1,014) Difference (95% CI)		P for hetero-geneity	Women (n=1,438) Difference (95% CI)		Men (n=1,029) Difference (95% CI)		P for hetero-geneity	None (n=633) Difference (95% CI)		Having any risk factor (n=1,832) Difference (95% CI)		P for hetero-geneity
Employment status															
Employed	Reference		Reference			Reference		Reference			Reference		Reference		
Unemployed	0.12	(−0.07 to 0.31)	−0.05	(−0.31 to 0.21)	0.222	−0.03	(−0.22 to 0.17)	0.00	(−0.25 to 0.25)	0.992	0.10	(−0.19 to 0.39)	−0.06	(−0.23 to 0.12)	0.198
Length of education															
≥13 years	Reference		Reference			Reference		Reference			Reference		Reference		
10–12 years	−0.15	(−0.32 to 0.02)	−0.15	(−0.48 to 0.18)		−0.13	(−0.34 to 0.08)	−0.28	(−0.51 to −0.05)		−0.29	(−0.57 to 0.00)	−0.16	(−0.35 to 0.03)	
<10 years	−0.37	(−0.64 to −0.11)	−0.59	(−0.92 to −0.26)	0.456	−0.61	(−0.88 to −0.34)	−0.59	(−0.88 to −0.30)	0.513	−0.83	(−1.28 to −0.37)	−0.56	(−0.78 to −0.34)	0.877
Marital status															
Ever married	Reference		Reference			Reference		Reference			Reference		Reference		
Never married	−0.22	(−0.44 to 0.01)	0.70	(−0.55 to 1.96)	0.126	−0.27	(−0.63 to 0.08)	−0.04	(−0.40 to 0.32)	0.323	−0.21	(−0.61 to 0.19)	−0.11	(−0.44 to 0.21)	0.438
Living status															
Living together	Reference		Reference			Reference		Reference			Reference		Reference		
Living alone	0.01	(−0.31 to 0.33)	−0.16	(−0.46 to 0.14)	0.417	−0.16	(−0.44 to 0.11)	−0.11	(−0.46 to 0.23)	0.864	−0.16	(−0.69 to 0.36)	−0.13	(−0.36 to 0.10)	0.975
Equivalent household expenditure															
Upper 4 quintiles	Reference		Reference			Reference		Reference			Reference		Reference		
Lowest quintile	−0.17	(−0.37 to 0.04)	−0.34	(−0.61 to −0.07)	0.297	−0.36	(−0.57 to −0.14)	−0.11	(−0.36 to 0.15)	0.146	−0.25	(−0.59 to 0.09)	−0.24	(−0.43 to −0.06)	0.967

CI, confidence interval.

“Having any risk factor” was defined as having at least one of hypertension, diabetes, hypercholesterolemia, low HDL cholesterol, or smoking.

Models include age (except for age groups) and sex (except for gender groups).
